# Ocular cysticercosis with vitreous hemorrhage: a rare complication of a common disease

**DOI:** 10.1186/s40064-015-1006-7

**Published:** 2015-05-07

**Authors:** Rajendra Singh Jain, Sunil Kumar, Indu Bhana, Rakesh Agarwal

**Affiliations:** Department of Neurology, Sawai Man Singh Medical College, Jaipur, Rajasthan India

**Keywords:** Ocular cysticercosis, Vitreous hemorrhage, Neurocysticercosis, Taenia solium

## Abstract

**Introduction:**

Cysticercosis, a helminthic infestation caused by *Taenia solium*, can produce central nervous system, muscles, visceral, subcutaneous tissues and skin manifestations. Ophthalmological involvement can affect eyelids, conjunctiva, anterior chamber, uvea, vitreous, retina, extraocular muscles and optic nerve. Simultaneous co-occurrence of intracranial and intraocular cysticercosis is a common presentation in clinical practice.

**Case description:**

We report a case of young girl who was diagnosed with multiple intracranial neurocysticercosis lesions and was on antiepileptic drugs, following which she presented with progressive painless vision loss from both the eyes. Indirect ophthalmoscopic examination showed evidence of subretinal cysts, retinal hemorrhage and retinal detachment in both the eyes. Surprisingly, bilateral vitreous hemorrhage was also detected. Ocular B-scan ultrasonography, orbital MRI and computed tomography revealed retinal detachment with vitreous hemorrhage in both the eyes. Magnetic resonance imaging (MRI) of brain showed multiple intraparenchymal small cystic lesions in bilateral cerebral and cerebellar hemispheres, basal ganglia, thalami and brainstem.

**Conclusions:**

Vitreous and retinal detachment are well known complications of intraocular cysticercosis, however, vitreous hemorrhage as preoperative feature has never been reported before, although vitreous hemorrhage as postoperative complication is common.

## Background

Cysticercosis caused by *Taenia solium* is endemic in developing countries like India due to poor hygiene and contamination of food and water (David and Mathai 2000). Co-infection of brain (neurocysticercosis) and eye (intraocular cysticercosis) is a common presentation in clinical practice. Intraocular involvement by cysticercosis is a feared complication due to high risk of vision loss. Though, vitreous and retinal detachment are well known complication of intraocular cysticercosis (Sohoni 2013), vitreous hemorrhage as a complication of intraocular cysticercosis has not been reported in literature. A high index of suspicion for intraocular cysticercosis should be kept when a patient with neurocysticercosis develops acute to subacute progressive vision loss or has history of travel to endemic area. Diagnosis should be confirmed by appropriate laboratory investigations like ELISA assay, ocular B-scan ultrasonography and ocular imaging (Guigon and Trepsat 2002). Early and prompt management of intraocular cysticercosis is required for better outcome.

## Case description

A 14-year-old girl had one episode of seizure five months prior to admission. She was diagnosed to be having multiple intracranial neurocysticercosis lesions and started on carbamazepine therapy. After three months of treatment, she noticed intermittent distortion of vision (metamorphopsia), seeing black spots and floaters in both the eyes. Subsequently, she developed progressive painless vision loss from both the eyes. There was no history of diabetes mellitus, hypertension or tuberculosis. Ophthalmological examination did not show orbital swelling, redness of eye, excessive lacrimation and other signs of ocular or orbital inflammation. There was no evidence of adnexal, orbital or extraocular muscle involvement. Intraocular pressure was normal on both sides. Though, the pupils were slightly dilated, reaction to light and accommodation was normal. Visual acuity was limited to perception of light in both the eyes. Bilateral detailed indirect ophthalmoscopic examination revealed evidence of subretinal cysts, retinal hemorrhage and retinal detachment. Surprisingly vitreous hemorrhage was also detected in both the eyes. Rest of the neurological examination was unremarkable.

**Figure 1:**
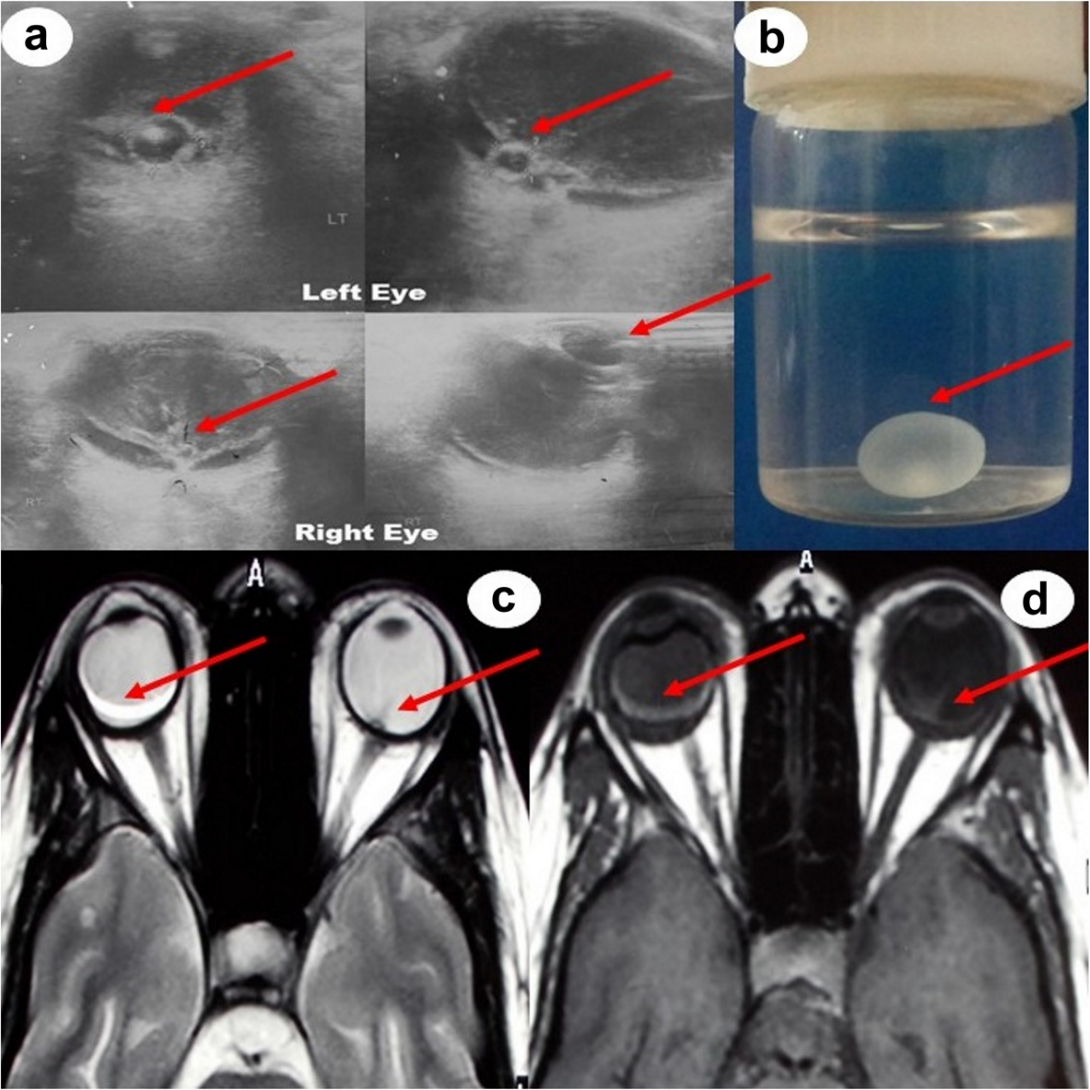
Ocular B-scan ultrasonography **(a)** showing septations and debris in vitreous on both sides. There is cystic area with small peripheral solid nodular area in subretinal space in both eyes (right and left eyes). [Arrows] Gross examination of pathological specimen revealed a thin walled, transparent, globular cyst with eccentrically placed small scolex **(b)**. [Arrow] Magnetic resonance imaging (MRI) of orbit showing V-shape retinal detachment in posterior chamber of left globe. Heterogeneous hyperintense signal is seen in posterior chamber of right globe **(c, d)** [Arrows].

**Figure 2:**
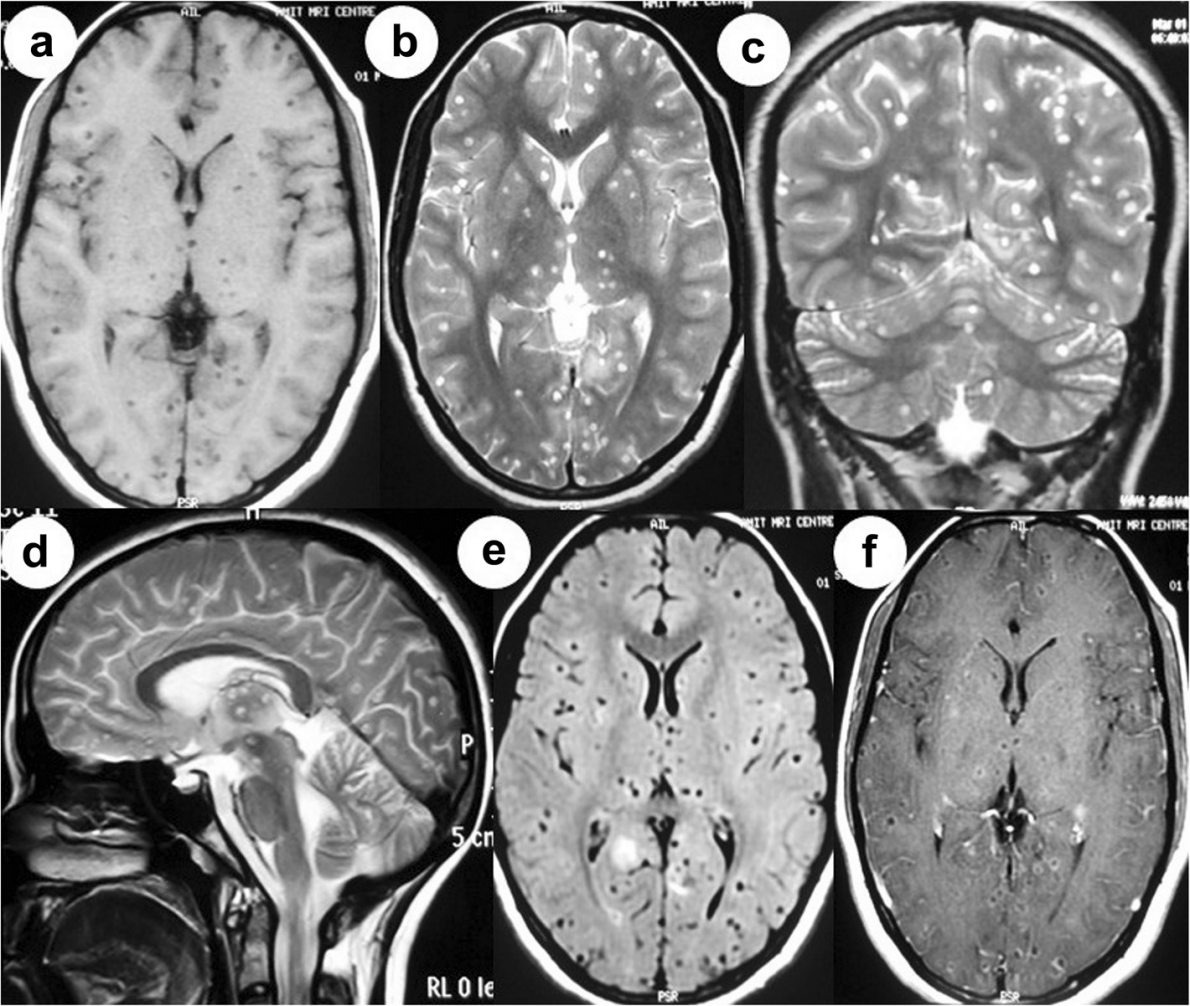
MRI brain showing multiple small cystic lesions in bilateral cerebral hemispheres, brainstem and bilateral cerebellar hemispheres with ring enhancement **(a-f)**.

Hemogram, serum biochemistry and x-ray chest were normal. Human immunodeficiency virus (HIV) was negative. Enzyme linked immunosorbent assay (ELISA) was strongly positive for Taenia solium. Ocular B-scan ultrasonography (USG) revealed septations and debris in vitreous in both eyes. It also showed cystic lesion with small peripheral solid nodular area in subretinal space in both eyes [Figure 1(a)]. Orbital computed tomography also depicted the vitreous hemorrhage with retinal detachment in both the eyes. Magnetic resonance imaging (MRI) of orbits showed heterogeneous hyperintensity in posterior chamber of eyes [Figure 1(c), (d)]. Ocular B-scan USG, orbital computed tomography and MRI orbit were consistent with vitreous hemorrhage and retinal detachment in both the eyes (right more than left). Subretinal cyst was located at the posterior pole of both the eyes. MRI brain revealed multiple small cystic intraparenchymal lesions in bilateral cerebral and cerebellar hemispheres, basal ganglia, thalami and brainstem. MRI T1-weighted image showed isointense to hypointense lesions. Fluid attenuated inversion recovery (FLAIR) and T2-weighted images showed hyperintense lesions with surrounding edema. Postcontrast T1-weighted image showed multiple ring enhancing lesions [Figure 2(a-f)].

A clinical diagnosis of vitreous hemorrhage due to ocular cysticercosis was made and it was further supported by various imaging modalities. The patient underwent retinal surgery for cyst extraction. A complete cyst excision was possible from right eye, but on left side, cyst got ruptured during intra-operative procedure. Gross examination of pathological specimen revealed a thin walled, transparent, globular cyst with eccentrically placed small scolex [Figure 1(b)]. Histopathological examination (stained with hematoxylin and eosin) showed the invaginated scolex with suckers, spiral canal and hooks thereby confirming the cysticercus cellulosae. A short course of oral steroid was given to the patient after cyst excision. At the time of discharge from hospital, she was able to count finger at one foot from right eye, but still had only perception of light from left eye. She was continued on carbamazepine (200 mg thrice a day) for seizures. At one-year follow up, seizures are well controlled. Visual acuity (Snellen chart) is 6/60 in right eye and complete loss of vision in left eye. Ophthalmoscopic examination shows partial retinal re-attachment and optic disc pallor in the right eye, whereas optic disc atrophy is present in left eye.

## Discussion and evaluation

Cysticercosis, a helminthic infestation caused by Taenia solium, can produce central nervous system, muscle, visceral, subcutaneous tissue and skin manifestations (Kaliaperumal et al. 2005). In eye, Taenia solium can affect any part from eyelids, conjunctiva, anterior chamber, uvea, vitreous, retina, extraocular muscles to even optic nerve (Pushker et al. 2001). Co-infection of neurocysticercosis and intraocular cysticercosis is a common presentation in clinical practice. The most common presentation of neurocysticercosis is seizures. The diagnosis of intraocular cysticercosis has special importance due to high risk of visual impairment. The possible mechanism of visual loss can be due to either compression from enlarging cyst or inflammatory reaction to cyst wall and toxic material released from dying cyst (Rath et al. 2010). The diagnosis of cysticercosis is based on clinical history, examination, serology and relevant imaging (Ziaei et al. 2011). If cyst can be excised, a detailed histopathological examination would be confirmatory.

Diagnosis of ocular cysticercosis need a high clinical suspicion and more dedicated investigations like ocular B-scan USG, CT orbit and MRI orbit (Jankharia et al. 2005). Our patient initially had intermittent black floaters and metamorphopsia and subsequently developed progressive painless loss of vision, which suggested vitreous and retinal pathology. A clinical diagnosis of vitreous hemorrhage with retinal detachment was made on ophthalmological examination; however, it was further supported by various imaging modalities prior to surgical intervention.

The patient had subretinal cysts, which is an uncommon location as compared to vitreous cysts. The parasite enters the retina through high flow choroidal circulation; subsequently migrates to the subretinal space and then into the vitreous. During the passage, it can cause cellular damage and inflammatory reaction to vessels, choroid and retina and subsequently produces chorioretinal scar and vascular damage. In our patient, vitreous hemorrhage may be related to rupture of damaged blood vessels due to severe inflammatory reaction.

Other causes of vitreous hemorrhage like trauma, proliferative diabetic retinopathy, hypertensive retinopathy, proliferative sickle cell retinopathy, Inflammatory conditions (Eales disease, Behcet’s disease and systemic lupus erythematosus) macroaneurysm, Terson syndrome, branch or central retinal vein occlusion, bleeding disorders and tumors were ruled out with appropriate clinical history and laboratory investigations (Spraul and Grossniklaus 1997).

Antihelminthic drugs like albendazole or praziquantel reduce the number of cysts and frequency of seizures in neurocysticercosis; however, they are unsatisfactory in management of subretinal cyst (Steinmetz et al. 1989). Early surgical excision of intraocular cysticercosis cyst is the treatment of choice (Steinmetz et al. 1989; Gemolotto 1955). If there is coinfection with intraocular and intracranial cysticercus, the complete intraocular cyst must be removed completely by surgery first, followed by cysticidal drugs and corticosteroids. Antihelminthic therapy is contraindicated in ocular cysticercosis because lysis and degeneration of intraocular cyst may induce intraocular inflammatory reactions and result in visual loss (Natarajan et al. 1999). Our patient had simultaneous coinfection of brain and eye alongwith vitreous hemorrhage, which is an extremely rare complication. Vitreous hemorrhage is a well-known complication of surgery during cyst excision (Sharma et al. 2003); however, it was detected preoperatively in our case. Unfortunately, in our patient, during surgical excision cyst got ruptured in left eye, which might have further added to vision loss. That may be because of intraocular inflammatory reaction to desiccated material resulting in retinal and optic disc damage. However, on right side vision improved after cyst excision.

## Conclusions

A high index of clinical suspicion is needed to diagnose ocular cysticercosis. Early and prompt management is required for better outcome. Though, vitreous and retinal detachment are well known complications of intraocular cysticercosis, vitreous hemorrhage (preoperative) has not been reported in literature. Our case highlights that vitreous hemorrhage may also be a complication of intraocular cysticercosis.

## Consent

Written informed consent was obtained from the patient’s guardian/parent/next of kin for the publication of this report and any accompanying images.

## Abbreviations

MRI: Magnetic resonance imaging
CT: Computed tomography
B-scan USG: B-scan ultrasonography
